# Association of aortic root diameter and vascular function with an exaggerated blood pressure response to exercise among elite athletes

**DOI:** 10.1007/s00392-024-02591-3

**Published:** 2024-12-19

**Authors:** Astrid Most, Vincent Groesser, Sophie Hoelscher, Rebecca Weber, Ebru Akdogan-Gernandt, Lutz Kraushaar, Oliver Dörr, Jamschid Sedighi, Stanislav Keranov, Faeq Husain-Syed, Christian W. Hamm, Samuel Sossalla, Pascal Bauer

**Affiliations:** 1 Department of Cardiology and Angiology, Justus Liebig University Giessen, 35390 Giessen, Germany; 2 Department of Internal Medicine II, Justus Liebig University Giessen, Giessen, Germany; 3 Adiphea GmbH, Werbach, Germany; 4 Department of Cardiology, Kerckhoff Heart and Thorax Center, Bad Nauheim, Germany; 5 German Center for Cardiovascular Research (DZHK), Partner Site Rhine-Main, Bad Nauheim, Germany

**Keywords:** SBP/MET slope, Elite athletes, Aortic root diameter, Exaggerated blood pressure, Vascular function, Central blood pressure

## Abstract

**Background:**

The systolic blood pressure/workload (SBP/MET) slope was recently reported to reliably identify an exaggerated blood pressure response (eBPR) in the normal population and in athletes. We investigated whether the aortic root diameter (AoD) also correlates with an eBPR and vascular function in elite athletes.

**Methods:**

We examined 652 healthy male elite athletes (age 25.8 ± 5 years) of mixed sports with a standardized maximum exercise test. Central blood pressure and vascular function were measured non-invasively with a validated oscillometric device. The SBP/MET slope was calculated and the threshold for an eBPR was set at > 6.2 mmHg/MET. Two groups were defined (≤ 6.2 and > 6.2 mmHg/MET), and an association between AoD and vascular function with the SBP/MET slope was evaluated for each group.

**Results:**

Athletes with an eBPR (*n* = 191, 29%) displayed a higher systolic central BP (103 ± 7.7 vs. 101 ± 9.2 mmHg, *p* = 0.004), larger AoD (32.8 ± 3.3 vs. 31.9. ± 3.2 mm, *p* < 0.001), a higher AoD/left ventricular end-diastolic diameter (LVEDD) ratio (0.62 ± 0.061 vs. 0.59. ± 0.056, *p* < 0.001), a lower LVEDD/AoD ratio (1.64 ± 0.16 vs. 1.69. ± 0.16, *p* < 0.001), and a lower absolute (299 ± 59 vs. 379 ± 65 W, *p* < 0.001) and relative workload (3.17 ± 0.55 vs. 4.05 ± 1.2 W/kg, *p* < 0.001) vs. athletes with a normal SBP/MET slope (*n* = 461, 71%). No differences between the two groups were found after indexing AoD to body surface area (BSA) (14.76 ± 1.36 vs. 14.73 ± 1.41, *p* = 0.772).

**Conclusion:**

Athletes with eBPR displayed altered AoD/LVEDD and LVEDD/AoD ratios, whereas AoD/BSA indexing was not different. Further longitudinal studies are encouraged to explore these metrics and their role in aortic remodeling of athletes.

## Introduction

The phenomenon of the “athlete’s heart”, characterized by functional and structural changes in the right and left ventricle, has been described in elite athletes participating in mixed and endurance sports disciplines [1–3]. However, the effects of intensive endurance training are not confined to the myocardium but extend to the vascular system, particularly the aorta [4–6]. Competitive athletes were found to have a larger aortic root diameter (AoD) and a larger ascending aorta than the general population [7–9], and differences were dependent on sex and type of sports [7, 9]. High-intensity sports were associated with larger aortic root size, with male endurance athletes displaying the largest diameters [7]. However, aortic root dilation above the currently defined upper limit of > 40 mm in male athletes is uncommon [5, 7, 9–11]. Aortic dilation is the principal risk factor for acute aortic syndrome [12], and the increase in systolic blood pressure (SBP) during training and competition may accelerate the progression of the dilatation; this also increases the risk of aortic dissection, which constitutes a rare cause of sports-associated sudden cardiac death in young adults [13]. The precise mechanism of the aortic root enlargement in athletic populations is unknown, but a combination of sex, body size, sporting discipline, and duration and intensity of training may be contributing factors, whereby the blood pressure response (BPR) to exercise is also assumed to be important [9, 14].

This latter assumption is of particular interest, since arterial hypertension is the most common cardiovascular disease in athletes [15, 16]. The underlying mechanisms are not fully understood, but it is believed that the high amount of training and longer exposure to higher exercise-induced BP levels may contribute to this phenomenon [17]. It was shown that individuals with an exaggerated BP response to exercise (eBPR), even with normal resting BP, are at increased risk of developing arterial hypertension and cardiovascular events in the future [18, 19]. This also applies to athletes [20–22], although data informing the definition of a normal or eBPR to exercise in athletes are scarce, despite clinical exercise testing being a key component of pre-participation screening [16]. The 2018 guidelines of the European Society of Cardiology (ESC) [23] and even the updated guidelines of the European Society of Hypertension [24] state that there is currently no consensus on a normal BPR during exercise.

Recently, a workload-indexed approach to classify the BPR to exercise in the general population was introduced by Hedman et al. [25] to define an eBPR. An SBP/MET slope, defined as the change in SBP in response to workload (metabolic equivalent of task, MET), greater than the threshold of 6.2 mmHg/MET in males was associated with a 27% higher risk of mortality over 20 years compared to those with an SBP/MET slope < 4.3 mmHg/MET [25]. The utility of the SBP/MET slope for pre-participation screening was demonstrated by our group for male [26, 27] and female elite athletes [28]. Furthermore, an eBPR, defined as an SBP/MET slope > 6.2 mmHg/MET, has been shown to predict left ventricular (LV) hypertrophy independently of age and sex in elite athletes referred for clinical evaluation in a pre-participation setting in Germany [22].

Central BP (CBP) and aortic pulse wave velocity (PWV) are believed to be better indicators of cardiovascular risk than brachial BP [29], as they are independently correlated with organ damage [30, 31]. In addition, previous studies have suggested that increased arterial stiffness precedes the development of hypertension, and CBP was found to be a significant predictor of new-onset hypertension [32] and of an eBPR to exercise in male elite athletes [33]. Since subclinical vascular impairment leads to an eBPR to exercise even in the absence of hypertension at rest [34–36], vascular functional assessment can provide additional information for cardiovascular risk classification, especially in “apparently healthy” and well-trained individuals. Recently, validated noninvasive oscillometric devices have been introduced to simplify clinical assessment of vascular functional impairment at rest [37].

In summary, information about vascular impairment and an eBPR to exercise in athletes may help to understand aortic root remodeling in athletes. In elite endurance and mixed-sports disciplines, a sustained increase in cardiac output and concomitant volume load on the aorta during exercise is accompanied by significant surges in systemic blood pressure, which may lead to a progressive enlargement of the aorta. This hypothesis is supported by recent studies that showed that about 30% of former elite American football players [38] and nearly 25% of master-level rowers and runners [39] displayed aortic dilation.

The AoD in athletes has not yet been evaluated with regards to the SBP/MET slope definition of an eBPR. Therefore, we compared the functional and structural adaptations of male athletes (participating in different high-intensity mixed sports) with an eBPR with athletes with a normal BPR. We hypothesized that the former would display an enlarged AoD and worse vascular function compared with the latter and that an eBPR would be linked to a larger AoD. Hence, we investigated the significance of correlations of AoD as well as the newly introduced AoD to LV diameter ratio [40] with vascular functional parameters and with the SBP/MET slope in male elite athletes. In addition, we assessed the influence of clinical and sports-related parameters as well as echocardiographic factors on these parameters in our group of healthy male elite athletes.

## Methods

### Study design

This was a single-center, cross-sectional registry study conducted at the University Hospital of Giessen involving professional athletes during the routine pre-season medical monitoring program of the first German handball and basketball divisions and the second German handball, soccer, and ice hockey divisions. Data were collected in July and August of the years 2017–2023 after a 6-week competition-free interval. Only male athletes aged 18–39 years were included in the study and athletes with known connective tissue disease were excluded.

Subjective health status, medication, nutrition supplementation, amount of training, and history of training were assessed by questionnaire. Only individuals free of underlying cardiovascular diseases and medication were included. All tests were conducted at least 3 h post-prandially, and subjects refrained from exercise for at least 36 h prior to the test.

All participants provided their written informed consent. The local ethics committee of the University of Giessen approved the study protocol (AZ 15/17). The study was performed in accordance with the ethical standards laid down in the Declaration of Helsinki and its later amendments.

### Study population

The study enrolled a cohort of 652 male professional athletes of different ethnicities who participated in various mixed-sports disciplines such as handball (*n* = 252), basketball (*n* = 179), ice hockey (*n* = 126), and soccer (*n* = 95) and who hailed from different countries. All participants were non-smokers, did not consume snus and did not take any medications or supplements regularly. A comprehensive physical examination, 12-lead electrocardiogram (ECG), resting BP measurements, and transthoracic echocardiography were performed on all individuals. The participants' age, height, weight, and body mass index (BMI) were recorded, and their body surface area (BSA) was calculated using the DuBois formula.

### Blood pressure measurement at rest

The study utilized a validated automatic device based on a standard sphygmomanometer technique (Boso clinicus, Bosch + Sohn GmbH & Co. KG, Germany) to measure resting brachial BP. The cuff used for measurements was adjusted to the individual’s arm circumference. A trained research associate performed measurements on both arms of the athlete while they were in a sitting position, following a resting period of 5 min, and repeated the measurements after 2 min. Athletes with a mean SBP ≥ 140 mmHg or diastolic BP (DBP) ≥ 90 mmHg were excluded from the study.

### Echocardiography

All athletes were examined by standard transthoracic echocardiography administered by an experienced cardiologist according to the current recommendations [41, 42] using a Philips cx50 echocardiography system (Philips, Eindhoven, The Netherlands) with the participant in a left lateral supine position. Standard measurements of cardiac dimensions, contractility, and diastolic function were obtained. Each parameter was assessed in three to five consecutive cardiac cycles, and mean values were used for data recording and analysis.

The AoD was measured from the 2D parasternal long-axis view at the center of the Valsalva sinuses, perpendicularly to the axis of the aorta, with the “leading-edge to leading-edge” technique, and averaged over five consecutive cycles. The value of the AoD was indexed to BSA and height (ratiometric and allometric scale) and, in addition, to left ventricular end-diastolic diameter (LVEDD) and vice versa. In deriving the allometric ratio, the ß exponent used was 1.025 (expressed as height^1.025^; C value 0.05).

LV wall thicknesses and diameters were evaluated in the parasternal long-axis view at the level of mitral valve coaptation. Further, volumes and LV ejection fraction (LVEF) were determined using Simpson’s biplane method. LV stroke volume was calculated as the product of LV outflow tract area and outflow tract time-velocity integral, and right ventricular (RV) stroke volume was calculated similarly as the product of RV outflow tract area and outflow tract time-velocity integral.

LV mass was calculated using the Devereux formula and indexed to BSA to obtain the LV mass index (LVMI). LV hypertrophy was defined as an LVMI > 115 g/m^2^. Left atrial volume index (LAVI) was obtained by the area-length method.

Peak tricuspid regurgitant velocity (TRV) was measured from the spectral profile of the tricuspid regurgitation jet in the RV inflow projection of the parasternal short-axis view or the apical four-chamber view. Pulmonary artery systolic pressure (SPAP) was then calculated based on the simplified Bernoulli equation applied to TRV by adding a value of right atrial pressure as measured by inferior vena cava respiratory index to the systolic trans-tricuspid gradient. SPAP was assumed to equate the RV systolic pressure in the absence of pulmonary stenosis and/or RV outflow tract obstruction.

Tricuspid annular plane systolic excursion (TAPSE) was measured from the four-chamber views by placing an M-mode cursor through the tricuspid annulus and measuring the excursion distance in mm between end-diastole and end-systole.

### Non-invasive assessment of vascular function

The vascassist2^®^ device, developed by isymed GmbH (Butzbach, Germany), was used to collect pulse pressure waveforms through oscillometry in a non-invasive manner. This device uses a validated model of the arterial tree consisting of 721 electronic circuits that mimic an individual’s pulse pressure waves by adjusting the circuits’ capacitance, resistance, inductance, and voltage [37, 43]. The system uses evolutionary algorithms to optimize the fidelity of the pulse pressure wave replication, ensuring that fidelity replications of 99.6% or higher are included in the analysis.

All participants underwent non-invasive vascular evaluation after resting for 15 min in a supine position. The evaluation was performed using four conventional cuffs adapted to the upper arm and forearm circumferences of each participant. Radial and brachial pulse pressure waves were obtained on both arms with step-by-step deflation of the cuffs. The measurements were conducted in a room with a comfortable and stable temperature of 22 °C and no external stress influences. Participants were instructed to remain still during the pulse pressure wave acquisition, and stable and valid results were ensured through the performance of two brachial and three radial measurements, with a 30-s break between each measurement phase. The total duration of the examination was 15 min.

The acquired pulse pressure waves were then analyzed with a validated electronic model of the arterial tree to assess vascular functional parameters. Brachial and radial SBP and DBP, CBP, PWV, augmentation index (Aix), augmentation index at a heart rate of 75 bpm (Aix@75), resistance index (R), total vascular resistance, and ejection duration were calculated. CBP was determined using a validated transfer function based on the peripheral arterial waveform, and calculation of Aix@75 was also based on the pulse waveform.

### Exercise testing

Athletes underwent a standardized progressive maximal cycling ergometer test while their brachial BP and ECG were automatically measured and recorded (Schiller AG^®^, Switzerland). The test began with a warm-up period of 2 min at 50 W that was followed by a load level of 100 W that was increased by 50 W every 2 min until exhaustion, which was defined as the participant’s inability to maintain the load for 2 min. The interpolation of the maximum workload when a stage was not fully completed has been calculated as followed:

$$\begin{aligned} {\text{W max}}. & = {\mathrm{W}}\;{\mathrm{last}}\;{\mathrm{completed}}\;{\mathrm{stage}} + \left( {{\mathrm{time}}\;{\mathrm{completed}}\;{\mathrm{in}}\;{\mathrm{incomplete}}\;{\mathrm{stage}}/{\mathrm{duration}}\;{\mathrm{of}}\;{\mathrm{the}}\;{\mathrm{stage}}} \right) \\ & \quad \times \Delta \;{\mathrm{Watt}}\;\left( {{\mathrm{stage}}\;{\mathrm{increase}}} \right). \\ \end{aligned}$$

The test ended with a decrease in load to 25 W for 3 min of active recovery, followed by a 2-min cool-down period at rest. The test concluded with a final ECG recording and a brachial BP measurement. BP was measured at every stage during test and recovery periods, including at the maximum workload, immediately after the maximum workload, immediately after the end of the test, and after 5 min of recovery. Heart rate was measured with continuous ECG recording throughout the test and recovery periods. The absolute maximum workload of the athletes as well as the workload adjusted to individual body weight were assessed. Other measurements included maximum heart rate and heart rate at rest and 5 min after the exercise test. Increases in SBP and DBP were calculated from peak and baseline (resting) values. Pulse pressure was calculated as SBP–DBP at rest and at maximum exercise. In addition, mean BP was determined as: DBP + (SBP–DBP)/3.

MET was estimated using standard equations for cycling ergometers [44]. The ΔSBP was calculated as (maximum SBP–SBP at rest) and indexed by the increase in MET from rest (ΔMET calculated as peak MET–1) to obtain the SBP/MET slope. Based on their SBP/MET slope, athletes were allocated to either of two groups: the first group was defined as normal BPR to exercise with SBP/MET slope ≤ 6.2 mmHg/MET, and the second group was classified as having an eBPR with SBP/MET slope > 6.2 mmHg/MET.

### Statistical analysis

Descriptive analyses were carried out on all study variables for the total sample and separated by SBP/MET slope (≤ 6.2 mmHg/MET and > 6.2 mmHg/MET). All data are presented as mean ± standard deviation (SD). The Shapiro–Wilk test was used to determine normal distribution. If the data were determined to have a skewed distribution, all analyses were performed on normalized data. Between-group comparisons were made using independent sample t tests.

Pearson correlation was used to assess the linear relationship between the SBP/MET slope and AoD and the newly introduced AoD/LVEDD index. SBP/MET slope was dichotomized as mentioned above to calculate Odds ratios using Fisher’s Exact Test. Effect size was calculated using Cohen’s *d*. The two-tailed significance level was set at *p* < 0.05 for all measurements. All statistical analyses were performed using the IBM SPSS Statistics for Macintosh, Version 27.0 (IBM Corp., Armonk, NY, USA).

## Results

### Cohort characteristics

All 652 male elite athletes included in the study were participants in mixed team sports disciplines that are characterized by a high-intensity level (handball, ice hockey, soccer and basketball) [16]. The mean age of the participants was age 25.8 ± 5.1 years; the mean height was 188.9 ± 8.4 cm and weight 91.7 ± 12.2 kg, resulting in a BMI of 25.6 ± 1.9 kg/m^2^. The probands were experienced athletes who had participated in professional training for 8.8 ± 5.1 years with a current mean training time of 18.6 ± 3.7 h/week.

Athletes with an SBP/MET slope ≤ 6.2 mmHg/MET displayed a lower body weight (90.6 ± 12.2 vs. 94.5 ± 11.5 kg, *p* < 0.001), BMI (25.5 ± 1.8 vs. 26 ± 2.1 kg/m^2^, *p* = 0.003), and BSA (2.14 ± 0.33 vs. 2.23 ± 0.17 m^2^, *p* < 0.001) compared to those with SBP/MET slope > 6.2 mmHg/MET (Table 1). Further, athletes with an SBP/MET slope ≤ 6.2 mmHg/MET had a significantly lower training volume per week (18.4 ± 4 vs. 19.4 ± 2.6 h/week, *p* < 0.001) compared to those with SBP/MET slope > 6.2 mmHg/MET. Further clinical characteristics, anthropometric data, and specific training data are displayed in detail in Table 1.

**Table 1:** Clinical characteristics of the cohort athletes according to defined SBP/MET slope cut-off

	Male elite athletes		
	SBP/MET slope ≤ 6.2 mmHg/MET	SBP/MET slope > 6.2 mmHg/MET	*p* value
Number (%)	461 (71)	191 (29)	
Age (years)	25.9 ± 5.1	25.4 ± 5	0.232
Height (cm)	188.4 ± 8.7	190.4 ± 7.5	**0.003**
Body weight (kg)	90.6 ± 12.2	94.5 ± 11.5	** < 0.001**
Body mass index (kg/m^2^)	25.5 ± 1.8	26 ± 2.1	**0.003**
Body surface area (m^2^)	2.14 ± 0.33	2.23 ± 0.17	** < 0.001**
Training history (years)	8.9 ± 5.2	8.6 ± 5.1	0.414
Training per week (h)	18.4 ± 4	19.4 ± 2.6	** < 0.001**
Systolic blood pressure (mmHg)	124.6 ± 11.5	124.7 ± 9.8	0.896
Diastolic blood pressure (mmHg)	63.8 ± 10.5	64.4 ± 10.5	0.103
Mean arterial blood pressure (mmHg)	80.2 ± 9.6	80.7 ± 9.5	0.508
Resting heart rate (beats/min)	58.7 ± 10.7	56.6 ± 9.6	**0.011**

Values are expressed as mean ± SD. Bold values denote statistical significance at the *p* < 0.05 level

The mean systolic and diastolic BP values of the entire study cohort were 124.6 ± 11 mmHg and 64 ± 9.6 mmHg, respectively. There were no differences in SBP between athletes with an SBP/MET slope ≤ 6.2 mmHg/MET (124.6 ± 11.5 mmHg) and those with an SBP/MET slope > 6.2 mmHg/MET (124.7 ± 9.8 mmHg). Athletes with an SBP/MET slope > 6.2 mmHg/MET displayed a lower heart rate at rest compared with their peers with an SBP/MET slope ≤ 6.2 mmHg/MET (56.6 ± 9.6 vs. 58.7 ± 10.7 bpm, *p* = 0.011).

### Vascular functional and central blood pressure measurements

There were no differences in aortic PWV, augmentation pressure, ejection duration, Aix@75, total peripheral resistance, mean aortic BP, or central DBP between the two groups (Table 2). In contrast, there were differences in central SBP, with higher values in athletes in the > 6.2 mmHg/MET group (102.9 ± 7.7 vs. 100.6 ± 9.2 mmHg, *p* = 0.004).

**Table 2:** Vascular function, echocardiographic, and exercise testing results of the cohort athletes according to defined SBP/MET slope cut-off

	Male elite athletes		*p* value
	SBP/MET slope ≤ 6.2 mmHg/MET	SBP/MET slope > 6.2 mmHg/MET	
*Central blood pressure and vascular function*			
Systolic central BP (mmHg)	100.6 ± 9.2	102.9 ± 7.7	**0.004**
Diastolic central BP (mmHg)	63.5 ± 9.9	64.4 ± 10.1	0.328
Mean aortic BP (mmHg)	78.6 ± 10.4	79.5 ± 10.1	0.508
Aortic pulse wave velocity (m/s)	6.3 ± 1.6	6.4 ± 1.4	0.385
Augmentation index @75 bpm (%)	− 21.1 ± 11.1	− 20 ± 11.2	0.293
Augmentation pressure (mmHg)	− 5.5 ± 4.3	− 5.2 ± 4.1	0.438
Ejection duration (ms)	300.2 ± 29.2	301.3 ± 25.7	0.793
Total peripheral resistance (dyn*s/cm^5^)	1341 ± 395	1414 ± 414	0.553
Pulse pressure amplification (mmHg)	25.64 ± 5.8	26.2 ± 6.2	0.396
*Echocardiographic parameters*			
LV ejection fraction (%)	66.2 ± 4.6	66.9 ± 4.5	0.079
LV stroke volume (ml)	92.7 ± 19.5	92.4 ± 16.4	0.865
LV end-diastolic diameter (mm)	53.6 ± 3.7	53.5 ± 3.8	0.790
LV end-systolic diameter (mm)	33.3 ± 3.4	32.9 ± 3.5	0.138
Left atrial diameter (mm)	35.2 ± 3.3	37.2 ± 3.3	**0.013**
Left atrial volume index (ml/m^2^)	24.5 ± 4	27.4 ± 4.1	** < 0.001**
Septal wall thickness (mm)	10.10 ± 1	10.26 ± 1.2	0.126
Inferior wall thickness (mm)	9.7 ± 0.9	9.9 ± 1.1	0.089
LV mass index (g/m^2^)	87.9 ± 25.2	89.9 ± 24.2	0.343
Relative wall thickness (%)	33 ± 8	34 ± 6	0.452
*E*/*A* ratio	1.88 ± 0.45	1.84 ± 0.43	0.349
*E*/*E*′ lateral	5.23 ± 1.3	5.35 ± 1.34	0.390
*E*/*E*′ medial	6.79 ± 1.2	6.66 ± 1.41	0.299
*E*/*E*′ average	6.2 ± 1	6.2 ± 1.1	0.322
RV diameter 1 (mm)	36.5 ± 6.2	37.2 ± 6.2	0.088
TAPSE/SPAP (mm/mmHg)	1.28 ± 0.33	1.34 ± 0.35	**0.043**
Aortic root size (mm)	31.9 ± 3.2	32.8 ± 3.3	** < 0.001**
Aortic root size/BSA (mm/m^2^)	14.73 ± 1.41	14.76 ± 1.36	0.772
Aortic root size/height (mm/m)	0.17 ± 0.015	0.18 ± 0.015	**0.015**
Aortic root size/height^1.025^ (mm/m^1.025^)	0.17 ± 0.016	0.18 ± 0.015	**0.015**
Aortic root size/LVEDD Index	0.59 ± 0.056	0.62 ± 0.061	** < 0.001**
LVEDD/aortic root size Index	1.69 ± 0.16	1.64 ± 0.16	** < 0.001**
*Exercise testing*			
Systolic blood pressure at rest (mmHg)	126.8 ± 11.4	129 ± 13.6	**0.006**
Diastolic blood pressure at rest (mmHg)	77 ± 8.2	75.8 ± 8.2	0.103
Heart rate at rest (bpm)	61.3 ± 10.8	60 ± 9	0.154
Absolute workload (W)	378.6 ± 64.5	298.5 ± 58.9	** < 0.001**
Relative workload (W/kg)	4.05 ± 1.2	3.17 ± 0.55	** < 0.001**
Peak energy expenditure (MET)	14.7 ± 4	12.2 ± 1.7	** < 0.001**
Max. systolic BP (mmHg)	188.9 ± 18.2	213.4 ± 14.9	** < 0.001**
Max. diastolic BP (mmHg)	83.3 ± 9.4	85.2 ± 8.8	**0.018**
Max. heart rate (bpm)	178.9 ± 9.9	179.2 ± 11.3	0.675
Max. heart rate (% of calculated max. heart rate)	92.9 ± 5.9	94.2 ± 5.8	0.085
Rating of perceived exertion (Borg scale)	18.4 ± 0.4	18.6 ± 0.8	0.512
SBP/MET slope (mmHg/MET)	4.53 ± 1.46	7.85 ± 1.33	** < 0.001**

Values are expressed as mean ± SD. Bold values denote statistical significance at the *p* < 0.05 level

*BP* blood pressure, *bpm* beats per min, *BMI* body mass index, *LV* left ventricle, *RV* right ventricle, *SPAP* systolic pulmonary artery pressure, *TAPSE* tricuspid annular plane systolic excursion, *LVEDD* left ventricular end-diastolic diameter

### Echocardiographic characteristics

Echocardiographic characteristics are summarized in Table 2. The two groups differed in left atrial diameter (*p* = 0.013), left atrial volume index (*p* < 0.001), and TAPSE/SPAP ratio (*p* = 0.043), with athletes assigned to the > 6.2 mmHg/MET group displaying higher values for these parameters.

### Aortic root size

**Fig. 1:**
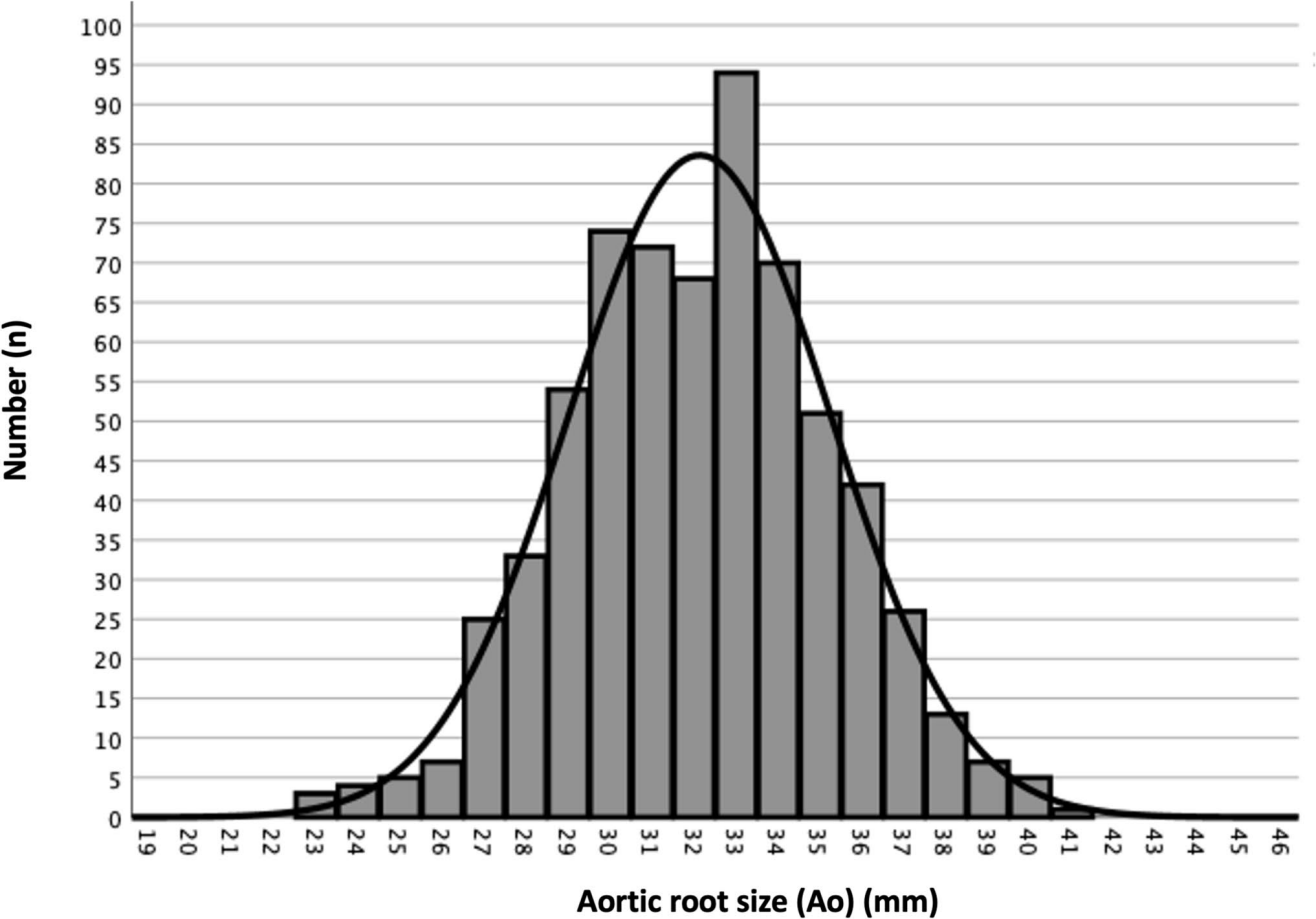
Aortic root dimensions in the investigated cohort of male elite athletes

The aortic root was larger in athletes belonging to the > 6.2 mmHg/MET group (AoD 32.8 ± 3.3 vs. 31.9 ± 3.2 mm, *p* < 0.001) compared to those in the SBP/MET slope ≤ 6.2 mmHg/MET group. Even after indexing AoD to height (*p* = 0.015), height^1.025^ (*p* = 0.015), and LVEDD (*p* < 0.001), these differences remained significant. Only after indexing AoD to BSA were no differences detected (*p* = 0.772). Detailed data are given in Table 2. Further, a histogram of the absolute AoD measures is shown in Fig. 1**.**

Overall, when applying the proposed absolute reference values of AoD for male athletes (40 mm), only seven athletes (1.1%) displayed values of 40 mm (five athletes) and above (two athletes, 41 mm). The maximum AoD measured was 41 mm. All athletes with an AoD of ≥ 40 mm had an SBP/MET slope of > 6.2 mmHg/MET and hence fell within the eBPR group.

### Exercise testing results

As expected, athletes with an eBPR displayed a higher SBP/MET slope compared with those with a normal BPR (7.85 ± 1.33 vs. 4.53 ± 1.46 mmHg/MET, *p* < 0.001). Further, these athletes had a higher SBP at the beginning of the test (*p* = 0.006) and a higher maximum systolic (*p* < 0.001) and diastolic BP (*p* = 0.018) compared with those with SBP/MET slope ≤ 6.2 mmHg/MET. In contrast, athletes with an eBPR achieved a lower absolute (*p* < 0.001) and relative (*p* < 0.001) workload and had, correspondingly, a lower peak energy expenditure (*p* < 0.001) compared with those with a normal BPR.

All athletes included in the study reached exhaustion during the exercise test. This was confirmed by multiple parameters, including the rate of perceived exertion (RPE), the percentage of calculated maximum heart rate, and the directly measured maximum heart rate. Importantly, there were no significant differences in all these parameters between athletes with an SBP/MET slope ≤ 6.2 mmHg/MET and those with an SBP/MET slope > 6.2 mmHg/MET. Detailed results supporting this are provided in Table 2 of the manuscript.

### Prevalence of eBPR

Application of a cut-off of 6.2 mmHg/MET for the SBP/MET slope to differentiate a normal from an exaggerated BPR resulted in the classification of 191 athletes (29%) as eBPR and 461 (71%) as normal BPR. Athletes with an eBPR displayed a maximum SBP of 213.4 ± 14.9 mmHg. In the eBPR group, the lowest maximum SBP was 193 mmHg and the highest was 264 mmHg, with range of SBP/MET slope from 6.3 to 9.8 mmHg/MET. In the group with a normal BPR, the lowest measured maximum SBP was 168 mmHg and the highest was 214 mmHg, respectively. The corresponding range in SBP/MET slope was 2.8–6.2 mmHg/MET.

There were no significant differences in the prevalence of an eBPR between the different investigated mixed-sports (Handball 28.7% (*n* = 72/252) vs. Basketball 29.6% (53/179) vs. Ice-hockey 29.8% (*n* = 38/126) vs. soccer 29.7% (*n* = 28/95).

### Correlation of SBP/MET slope with aortic root size and AoD/LVEDD index

The Pearson correlation coefficient between AoD and SBP/MET slope was calculated with *r* =  − 0.1407. The *p* value for this correlation is 0.7377, indicating that the relationship is not statistically significant at conventional levels (e.g., *p* < 0.05). This suggests that there is no strong linear relationship between AoD and SBP/METslope in the given data.

Additional statistical analyses were performed. Pearson correlation was used to assess the linear relationship between the SBP/MET slope and the AoD/LVEDD index. Pearson correlation coefficient was *r* = 0.1468 (*p* = 0.002), indicating a weak positive correlation between the SBP/MET slope and the AoD/LVEDD index.

Athletes with an SBP/MET slope > 6.2 mmHg/MET showed significantly higher AoD/LVEDD indices (*t* = − 4.2511, *p* < 0.0001). The Odds ratio of 1.6869 (*p* = 0.0018) indicates that athletes with an SBP/MET slope > 6.2 mmHg/MET are more likely to have above-median AoD/LVEDD values. The effect size (Cohen’s *d* = 0.3461) suggests a small to moderate practical significance.

## Discussion

Our study is the first to comprehensively evaluate aortic root size using different, newly introduced indices (“golden ratios”), CBP, and cardiovascular function in healthy male elite athletes participating in various team sports and to examine its correlation with the workload-indexed BP response to a standardized exercise test, measured as the SBP/MET slope. Our main findings are:

1. athletes with an eBPR displayed a larger AoD, even after indexing to height and LVEDD (“golden ratio”), compared with athletes with a normal BPR;
2. athletes with an eBPR displayed a significantly higher systolic CBP, but not diastolic CBP or brachial BP, compared with athletes with a normal BPR;
3. only 1.1% of the participants displayed an AoD ≥ 40 mm.

The relationship between exercise-induced hemodynamic responses and cardiovascular adaptations in elite athletes has been a subject of significant interest. As opposed to the well-known cardiac adaptations, leading to the term “athlete’s heart” [1], fewer studies have addressed the vascular adaptations [45–48]. In highly trained athletes, consistently larger aortic root dimensions compared to sedentary controls were found [8, 9, 49], but they rarely exceed the upper limits of normality [9]. Besides the well-known influencing factors of sex, age, height, and BSA, sporting discipline was identified as a relevant factor in athletes [7, 9, 16, 50]. In athletic populations, sporting disciplines with a high dynamic component, represented as mixed and endurance sports, were associated with the largest AoD values [5, 7, 49, 51].

The impact of these findings remains unclear: one study reported no progression of aortic root enlargement in young and highly trained athletes [52], whereas another found an increase in the aortic root size during an 8-year follow-up [14]. Recently, reports about the progression of aortic root enlargement in long-term endurance athletes [39] and former elite rugby players [53] have fuelled interest in this topic, raising concerns about aortic complications [12, 54, 55].

The precise mechanisms underlying the observed association warrant careful consideration. Repetitive exposure to elevated blood pressure during intense training and competition may induce structural adaptations in the aorta. Over time, this mechanical stress can result in structural adaptations, including alterations in extracellular matrix composition and smooth muscle cell proliferation, ultimately contributing to aortic dilation [52]. Also, genetic predispositions could play a role in this maladaptation, even in the absence of specific conditions like Marfane syndrome [2, 5, 16, 45].

Hence, characterization and identification of an eBPR during exercise is of particular interest in the pre-participation evaluation of athletes. In recent studies, the cut-off value > 6.2 mmHg/MET for the metric SBP/MET slope has emerged as a reliable indicator of an eBPR during exercise in the normal population [25]. Our previous studies have shown that the SBP/MET slope could also be used in pre-participation screening for elite male [26] and female [28] athletes to identify athletes at risk of an eBPR. In addition, Keller et al. [22] found that an SBP/MET slope > 6.2 mmHg/MET was associated with concentric remodelling and concentric hypertrophy of the left ventricle, higher LV mass, and larger left atrial area. In line with these findings, the higher left atrial volume index and central SBP in athletes identified as having an eBPR in our study suggests that higher hemodynamic stress during exercise may be the connecting link of this associations.

Central hemodynamic parameters and vascular function are known to play a crucial role in BP regulation [45, 56–62] and physical performance [63–65], not only in the general population [66] but also in athletes of different sports [48, 67, 68]. Although CBP is considered to be a more significant predictor of cardiovascular outcomes than brachial BP [29, 31, 32, 59, 69, 70], there are currently no normative values for CBP in athletes [45, 71].

An eBPR might be a reflection of an early functional vascular impairment, even in the absence of hypertension at rest [34, 36]. Similarly, a study by Haarala et al. [35] found that arterial stiffness, measured as PWV at rest, was able to predict an eBPR in young and healthy individuals. In contrast to this latter study, but in line with our previous findings in elite athletes [27], our current findings did not show any differences in PWV between the two groups. Our study cohort consisted of professional athletes who had a lower PWV, and Haarala et al. [35] did not present their data separately for males and females, which limits a comparison with our own results. Hence, our measured PWV values were consistent with previous studies and meta-analyses that analysed elite athletes of different sports [62, 67, 72, 73].

The development of hypertension in the general population has been shown to be predicted by the CBP [32]. This is a clinically relevant finding, as an eBPR is considered to be a precursor of future arterial hypertension [20]. Given the high training levels of our cohort athletes, the repeated exposure to an eBPR over time and during an athletic career may be cumulative and lead to hypertension-related organ damage and aortic enlargement [51]. Notably, our study cohort displayed normal brachial BP values at rest without differences between the two groups. However, we observed significantly higher systolic CBP in athletes with an eBPR than in those with a normal BPR.

An eBPR may be clinically relevant not only for future risk prediction but also for current performance levels [74]. Recent data from over 1000 younger (mean age 21 years) German competitive athletes showed that an eBPR, defined by an SBP/MET slope > 6.2 mmHg/MET, was associated with significantly lower VO2max (47.5 vs. 41.8 ml/min/kg) [22]. While our study did not measure VO2max as a performance marker, we acknowledge its importance and encourage future research to explore its relationship with the SBP/MET slope and physical performance in athletes.

Interestingly, despite only a minority of athletes displaying AoDs of ≥ 40 mm in our study and others, aortic root remodelling in football players [51] and enlargement in long-term endurance athletes [39] have been frequently reported. Hence, defining aortic root enlargement only based on absolute values seems inappropriate. Usually, the AoD is indexed for BSA or height; however, this method assumes a linear correlation between the values and the anthropometric characteristics that does not exist. In contrast, there is a non-linear relationship between the aortic size and BSA, with a plateau in tall individuals, as shown for BSA > 2.3 m^2^ and also height > 189 cm in men.

Recently, indexing aortic root size to LVEDD or vice versa was reported to have a greater power to differentiate between pathologic and physiologic remodelling in athletes, also in comparison to other “established” indexed parameters (AoD/BSA, AoD/height, AoD/height^1.025^) [40]. Our results support this assumption, and the values we obtained are consistent with the reported reference values [40] for the AoD/LVEDD index (our study: 0.59 ± 0.056 vs. reference for mixed-sports athletes: 0.59 ± 0.06) and the LVEDD/AoD index (our study: 1.69 ± 0.16 vs. reference for mixed-sports athletes: 1.71 ± 0.15).

These results reveal a compelling link between an eBPR in male elite athletes and a larger aortic root. Even after adjusting for the newly introduced aortic root/LV diameter “golden ratios,” the association between an eBPR and an altered AoD metrics remains noteworthy. Further, we observed a weak positive correlation between the SBP/MET slope and the AoD/LVEDD index. Specifically, those with an eBPR demonstrated significantly greater AoD/LVEDD values. The Odds ratio of 1.68 (*p* = 0.0018) indicates that athletes with an SBP/MET slope > 6.2 mmHg/MET are more likely to have above-median AoD/LVEDD values. Although the correlation remains weak, our findings raise the possibility that an eBPR in elite athletes may contribute to long-term aortic maladaptation. From a pathophysiologic standpoint, this would make sense and also may explain the reported progression of aortic root enlargement over time [39, 51, 52], even after ending of an active career [38, 53]. Therefore, as the newly introduced “golden ratios” have proven to be able to differentiate between physiologic and pathologic aortic remodelling in athletes, we suggest that these markers, instead of the other proposed indexed ratios, might be a more powerful tool to diagnose a pathologic aortic condition in athletes. Particularly in elite athletes in whom the condition is likely to be exacerbated by intensive training, a closer follow-up should be implemented and further investigations should be undertaken in the event of pathologic findings.

Understanding the association between an eBPR and aortic root dimensions has significant clinical implications. Monitoring and managing the BPR during training and competition may be crucial for preventing long-term cardiovascular consequences in elite athletes. In addition, identifying individuals with an increased risk of aortic maladaptation based on their hemodynamic response profiles could guide personalized interventions and surveillance strategies.

In conclusion, based on our study results, we propose that the newly introduced “golden ratios” should be used to differentiate physiologic from pathologic remodelling in male elite athletes. Further, exercise hypertension may be a crucial factor for developing aortic maladaptation and progression of aortic root remodelling in athletes. To identify athletes at risk who have an eBPR during exercise testing, the use of the SBP/MET slope with a cut-off value of > 6.2 mmHg/MET to define an eBPR is encouraged.

### Limitations and strengths

Our study has several limitations. A major limitation of the current study is the use of maximal heart rate and RPE to evaluate exhaustion during cycling ergometry. Both parameters have inherent variability and limitations, particularly in elite athletes. Advanced methods such as spiroergometry, which utilize parameters like a plateau in the *V*O_2_ curve and RER, were not performed in this study. This decision reflects the context of routine pre-participation screenings, which typically prioritize workload-indexed metrics and practical clinical applicability over maximal performance assessment. Future studies should consider incorporating spiroergometry to explore the relationship between physical performance and metrics like the SBP/MET slope in greater detail.

The number of participants limited its statistical power to reveal other associations or to determine diagnostic thresholds. The focus on male elite mixed-sports athletes might limit extrapolation of the results to other sport disciplines, to an older age group, or to women. Further, we did not control for diet and body composition. However, we included male elite athletes of a narrow age span who did not use medication and who were free of cardiovascular diseases, and we controlled for confounders like prior prolonged exercise sessions. Furthermore, the homogeneous study cohort and the rigid design of measuring cardiovascular function must be mentioned, which strengthens our analysis.

## Conclusion

Male elite athletes with an eBPR, defined as an SBP/MET slope > 6.2 mmHg/MET, displayed a larger AoD, a higher absolute maximum BP, and a lower performance compared to athletes with a normal BPR. Indexing aortic root size to LVEDD and vice versa (AoD/LVEDD and LVEDD/AoD, the “golden ratios”) revealed differences between athletes with an eBPR and those without. This was not the case when AoD was indexed to BSA. An eBPR might be a crucial factor for developing aortic maladaptation and for the progression of aortic root enlargement in male elite athletes. To identify athletes at risk who have an eBPR during exercise testing, we propose using the SBP/MET slope with a cut-off value of > 6.2 mmHg/MET to define an eBPR.

## Data Availability

The manuscript data will not be deposited.
